# The energetic consequences of behavioral variation in a marine carnivore

**DOI:** 10.1002/ece3.3983

**Published:** 2018-04-02

**Authors:** Elizabeth A. McHuron, Sarah H. Peterson, Luis A. Hückstädt, Sharon R. Melin, Jeffrey D. Harris, Daniel P. Costa

**Affiliations:** ^1^ Department of Ecology & Evolutionary Biology University of California Santa Cruz Santa Cruz CA USA; ^2^ Institute of Marine Sciences Long Marine Laboratory University of California Santa Cruz Santa Cruz CA USA; ^3^ Marine Mammal Laboratory Alaska Fisheries Science Center/NOAA Seattle WA USA

**Keywords:** California sea lion, doubly labeled water, field metabolic rate, foraging behavior, intraspecific variation, pinniped

## Abstract

Intraspecific variability in foraging behavior has been documented across a range of taxonomic groups, yet the energetic consequences of this variation are not well understood for many species. Understanding the effect of behavioral variation on energy expenditure and acquisition is particularly crucial for mammalian carnivores because they have high energy requirements that place considerable pressure on prey populations. To determine the influence of behavior on energy expenditure and balance, we combined simultaneous measurements of at‐sea field metabolic rate (FMR) and foraging behavior in a marine carnivore that exhibits intraspecific behavioral variation, the California sea lion (*Zalophus californianus*). Sea lions exhibited variability in at‐sea FMR, with some individuals expending energy at a maximum of twice the rate of others. This variation was in part attributable to differences in diving behavior that may have been reflective of diet; however, this was only true for sea lions using a foraging strategy consisting of epipelagic (<200 m within the water column) and benthic dives. In contrast, sea lions that used a deep‐diving foraging strategy all had similar values of at‐sea FMR that were unrelated to diving behavior. Energy intake did not differ between foraging strategies and was unrelated to energy expenditure. Our findings suggest that energy expenditure in California sea lions may be influenced by interactions between diet and oxygen conservation strategies. There were no apparent energetic trade‐offs between foraging strategies, although there was preliminary evidence that foraging strategies may differ in their variability in energy balance. The energetic consequences of behavioral variation may influence the reproductive success of female sea lions and result in differential impacts of individuals on prey populations. These findings highlight the importance of quantifying the relationships between energy expenditure and foraging behavior in other carnivores for studies addressing fundamental and applied physiological and ecological questions.

## 1 INTRODUCTION

Energy expenditure is central to understanding physiological and ecological processes. Body size is an influential factor affecting energy expenditure, although other factors, such as phylogeny, trophic level, ambient temperature, and behavior can also contribute to inter‐ and intraspecific differences in energy expenditure (Anderson & Jetz, 2005; Nagy, 2005). Foraging is one of the most energetically expensive behaviors for nonherbivorous species, resulting in high field metabolic rates (FMR) during search, pursuit, and capture of prey (Acevedo‐Gutiérrez, Croll, & Tershy, 2002; Gorman, Mills, Raath, & Speakman, 1998; Williams et al., 2014). Foraging behaviors themselves are not necessarily energetically equivalent because of prey behavior, although species that use more costly behaviors may experience greater energy gains following the principles of optimal foraging theory (Anderson & Karasov, 1981; Nagy, Huey, & Bennett, 1984). Within a species, changes in foraging effort in response to food limitation affect FMR (Bryant & Tatner, 1991; Costa, 2008), but it is less well understood how intraspecific variability in foraging behavior influences energy expenditure and acquisition.

Ecologists have long recognized the existence of intraspecific variability in foraging behavior, yet this variation was largely ignored as “noise” in ecological studies. In the last two decades, the assumption that individuals are ecological equivalents has been largely invalidated by studies showing that individual differences in foraging behavior are widespread and can be ecologically important, even for species that are generalists at the population level (Araújo, Bolnick, & Layman, 2011; Bolnick et al., 2003, 2011). Intraspecific variation in foraging behavior is often manifested through dietary differences, but also may reflect variation in habitat use, search tactics, or foraging strategies that are independent of diet (Ceia & Ramos, 2015). Because the end result of foraging is energy expenditure and acquisition, the ability to quantify the relationships between energy expenditure and behavior is a crucial component in understanding the physiological and ecological consequences of variability in foraging behavior. This is particularly important for carnivores because they have high energy requirements that place considerable pressure on prey populations (Carbone, Teacher, & Rowcliffe, 2007; Smith, Link, Cadrin, & Palka, 2015; Williams, Estes, Doak, & Springer, 2004), and are often important in structuring ecological communities (Ripple et al., 2014). Concurrent measurements of FMR and behavior are lacking for most large carnivores due to the challenge of obtaining estimates of FMR from free‐ranging animals.

Female fur seals and sea lions (otariids) are good model species to examine questions about the interplay between energy expenditure and behavior in large carnivores. All female otariids share similar reproductive characteristics—during lactation they are central‐place foragers, alternating short foraging trips to sea (days to weeks) with periods of onshore nursing at the rookery (Costa, 1991). This behavior makes them a tractable group for metabolic studies, as the commonly used method of doubly labeled water requires an individual to be captured twice across a relatively short time interval (Speakman, 1997). Central‐place foragers also place considerable pressure on local prey populations, which can result in resource limitation and high levels of competition (Elliott et al., 2009; Kuhn, Baker, Towell, & Ream, 2014). Individual variation in foraging behavior has been increasingly documented for female otariids, which may be a mechanism to reduce competition (Kernaléguen, Arnould, Guinet, & Cherel, 2015; Villegas‐Amtmann, Jeglinski, Costa, Robinson, & Trillmich, 2013). In particular, many populations use multiple foraging strategies typically characterized by differences in dive types (pelagic vs. benthic dives), dive depth, and location (Baylis et al., 2015; Kernaléguen et al., 2016; Villegas‐Amtmann, Costa, Tremblay, Salazar, & Aurioles‐Gamboa, 2008), with individual fidelity to a given strategy often maintained across multiple years (Arthur et al., 2015; Chilvers & Wilkinson, 2009). Interspecific comparisons of energy expenditure in free‐ranging otariids indicate that benthic‐diving species often have higher at‐sea FMRs and are more likely to exceed their calculated aerobic dive limit than pelagic‐foraging species, leading to the hypothesis that benthic diving is an energetically expensive strategy (Costa, Kuhn, Weise, Shaffer, & Arnould, 2004). Intraspecific variation in specific behaviors also appears to affect energy expenditure at sea, such as variability in dive depth (Costa & Gales, 2000) and the proportion of time diving (Arnould, Boyd, & Speakman, 1996). Despite the tractability of female otariids for metabolic studies, there remain relatively few studies that concurrently measure energy expenditure and foraging behavior.

The goal of our study was to determine how intraspecific variation in diving behavior affects energy expenditure in California sea lions (*Zalophus californianus*), an otariid that inhabits the dynamic California Current Ecosystem and uses foraging strategies that encompass all three of the diving patterns (epipelagic, mesopelagic, and benthic) characteristic of air‐breathing marine predators (McHuron et al., 2016). These diving patterns largely describe the position within the water column where the animal forages, which largely reflect prey type. We concurrently measured FMR and behavior of adult female California sea lions across a foraging trip using doubly labeled water and animal‐borne instruments (bio‐loggers) to (1) determine the influence of behavioral variation on at‐sea FMR and how foraging strategy influenced these relationships, (2) determine whether at‐sea FMR differed between foraging strategies, and (3) examine the relationship between energy expenditure and acquisition.

## 2 MATERIALS AND METHODS

### 2.1 Capture and instrumentation

Adult female California sea lions were captured at San Nicolas (*n *=* *10; SNI) and San Miguel Islands (*n *=* *6; SMI) in November and December of 2014. The majority of females were observed nursing a pup (*n *=* *13); the remaining females were lactating at the time of capture but were not observed with a pup. Once captured in a net, females were weighed (±0.1 kg), physically restrained, and anesthetized using gas anesthesia alone or in conjunction with an intramuscular injection of midazolam (0.15 to 0.20 mg/kg) administered with atropine (0.02 mg/kg).

Sea lions were instrumented with satellite tags and time‐depth recorders (TDR; Wildlife Computers, Redmond, WA), and a VHF tag (Advanced Telemetry Systems, Isanti, MN). All tags were mounted on a neoprene base, attached to high‐tension mesh netting using cable ties, and affixed to the dorsal midline using a quick‐setting epoxy. The combined package weights were <1% of body mass and approximately 2% of the cross‐sectional area. Sea lions were recaptured after one foraging trip when possible to remove instruments and collect blood samples.

### 2.2 Field metabolic rate (FMR)

We used the doubly labeled water method to estimate FMR (Nagy, 1980; Speakman, 1997), which relies on changes in oxygen and hydrogen isotopes in the blood over time to estimate CO_2_ production. This method, which has been validated against measures of food intake and O_2_ consumption for pinnipeds (Costa, 1987; Dalton, Rosen, & Trites, 2014; Sparling, Thompson, Fedak, Gallon, & Speakman, 2008), provides an integrated estimate of energy expenditure across the measurement period. These measurements can also be used to calculate water influx, which can be used as a proxy for food intake as sea lions generally do not drink seawater (Costa, 1987).

An initial blood sample was collected from the caudal gluteal vein into a tube containing no additives to determine background isotope levels. This was followed by a single injection of a weighed dose of sterile saline solution containing 99.8% ^2^H and either 97% (~19 ml; SNI) or 10% ^18^O (~130 ml; SMI). This resulted in >200 ppm enrichment above background ^18^O levels for all individuals (range of 207–327 ppm), which is well above enrichment levels recommended by Speakman (1997). Sea lions were held for 3–4 hr postinjection to allow the isotope to equilibrate in the body water space (Costa, 1987) after which a blood sample was collected to determine the equilibration isotope concentrations. A final blood sample was collected at recapture to determine the final isotope enrichment. Serum and stock isotope solution samples were stored frozen at −20°C in parafilm‐wrapped plastic internal‐threaded cryovials with an O‐ring to prevent evaporation.

Serum and stock solution samples were analyzed for isotope concentrations by Metabolic Solutions Inc. (Nashua, NH). Mean isotope concentrations from triplicate measurements were used in the calculation of CO_2_ production. Isotope dilution spaces were calculated using the plateau (initial) and scaling (final) methods (Speakman, 1997). There are a variety of equations that can be used to calculate CO_2_ production, and the different equations affect energy estimates (Speakman & Hambly, 2016). We chose the two‐pool Speakman, Nair, and Goran (1993) equation because it most closely approximates energy expenditure of otariids (Boyd, Woakes, Butler, Davis, & Williams, 1995; Dalton et al., 2014), but we also present estimates calculated using the Nagy (1980) equation for comparison with other studies. A value of 23.6 kJ/L CO_2_ was used to convert CO_2_ production to energy consumption (Costa, 1987; Costa, Antonelis, & Delong, 1991). Water influx was calculated using equations 5 and 6 in Nagy and Costa (1980) and the dilution spaces determined from ^18^O. As recommended by Speakman (1997), we also estimated the variability in our estimate of CO_2_ production as an indication of the precision of our estimate of FMR (Appendix S1). The resulting estimate of FMR represents time spent at sea and variable amounts of time onshore; we used the approach described in Costa and Gales (2003) to correct for onshore time and estimate at‐sea FMR.

### 2.3 At‐sea behavior

Satellite tags collected either GPS and/or ARGOS locations. Location data were filtered using a custom speed and angle filter to remove erroneous locations (>12 km/hr or >160°, Y. Tremblay). Hourly at‐sea locations were predicted using a continuous correlated random walk (R package, *crawl*, Johnson, London, Lea, and Durban (2008); ARGOS) or linear interpolation (GPS). Dive data were analyzed using a custom‐built program in MATLAB (IKNOS, Y. Tremblay). Dives were defined as any dive ≥4 m that lasted a minimum of 16 s. Dive data were used to calculate 14 variables that described the diving behavior and effort of each sea lion. These included characteristics of individual dives (e.g., mean depth), dive bouts (e.g., mean bout duration), and the foraging trip itself (e.g., percentage of time spent diving; Table S1). Dive data were also used to classify the foraging trip (trips >6 hr) of each sea lion to one of the three foraging strategies used by female California sea lions using methods presented in McHuron et al. (2016), which consisted of a principal components analysis (PCA) of 14 diving variables followed by a hierarchical cluster analysis to identify foraging strategies (Table S2). These 14 variables were generally different from those described in Table S1, focusing on behavioral variation at a finer scale that did not necessarily integrate behavior across the entire foraging trip (e.g., mean day depth instead of mean depth). There was a strong focus on variables that described the position within the water column where a sea lion foraged (epipelagic, mesopelagic, benthic) and differences in day and night behavior, as these variables may reflect differences in prey type. We calculated these 14 variables for each foraging trip and predicted the PCA scores and resulting foraging strategy using a linear discriminant analysis. If a female had more than one foraging trip to sea, we assigned her to a single strategy based on the dominant strategy across all foraging trips.

### 2.4 Statistical analyses

Diving variables were strongly correlated with each other, precluding the ability to include multiple behavioral variables within the same regression model. Instead, we used principal components regression, where the variables of interest are first input into a PCA to generate a reduced set of uncorrelated variables that can then be regressed against at‐sea FMR. Prior to analysis, we reduced our initial 14 variables to a smaller subset using exploratory plots of at‐sea FMR vs. each behavioral variable (Figure S2); a core assumption of this approach is that the direction(s) in which the behavioral variables show the most variation is the same as the variation in at‐sea FMR. The variables included in the analysis were mean dive duration, dive depth, bout duration, descent rate, and ascent rate. Varimax rotation of the variable loadings was used for interpretation of each principal component axis. Foraging strategy was included as a factor in the regression analysis to determine if foraging strategy had any influence on the relationship between these variables and at‐sea FMR.

Mean values of variables that represent behavior across the entire foraging trip are generally assumed to be the most appropriate to examine the relationships with at‐sea FMR because the doubly labeled water method produces a single value that integrates all at‐sea behavior. As mentioned above, these variables may not capture fine‐scale variation in foraging behavior that has biological relevance. We explored whether there were any patterns in at‐sea FMR and behavioral variation (as quantified by the foraging strategy analysis) to determine if sea lions that exhibited similar at‐sea behavior also had similar at‐sea FMRs. To accomplish this, we used a Mantel test to determine the correlation between distance matrices of the PCA scores from the foraging strategy analysis and at‐sea FMR (R package *vegan*, Oksanen et al., 2017). PCA scores were weighted based on the variability explained by each dimension before the distance matrix was created.

We used Pearson's correlations between at‐sea FMR and water influx rate to assess whether sea lions that expended more energy obtained a greater energetic gain. The reliability of water influx as a proxy for prey intake of sea lions in our study was assessed using a Pearson's correlation between the rate of mass change and water influx rate.

All statistical analyses were performed using R v.3.4.1. (R Core Group 2017). Mean values are shown ± *SD* unless otherwise stated.

## 3 RESULTS

Metabolic rate measurements were obtained for nine sea lions from SNI and all six sea lions from SMI (Table 1). The mean measurement interval was 9.5 ± 3.5 days; 11 sea lions were recaptured after one foraging trip, one after two foraging trips (C16), and three after 5+ trips (Supplemental Text). Field metabolic rates ranged from 1.52 to 5.48 W/kg with mean values of 3.90 ± 1.24 (SNI) and 3.48 ± 0.48 W/kg (SMI; Table 1). Females spent between 47% and 83% of the measurement interval at sea, resulting in estimated at‐sea FMRs of 3.29 to 6.97 W/kg (Table 2) with similar values between the two islands (SNI* *=* *5.45 ± 1.08 W/kg, SMI = 4.92 ± 0.59 W/kg; one‐way ANOVA, *F*_1,13_ = 1.14, *p *=* *.31). The large variability among sea lions in time spent at sea during the measurement interval was because of variable amounts of time spent ashore at the rookery and time ashore at other haul‐out sites during foraging trips.

**Table 1:** Mass, pup status and pup mass, measurement interval, time at sea, total body water (TBW), water influx, and estimates of CO_2_ production and field metabolic rate (FMR) of 15 adult female California sea lions from San Nicolas and San Miguel Islands

Sea lion ID	Initial mass (kg)	Final mass (kg)	Pup	Pup mass (kg)	Interval (days)	Time at sea (days)	TBW (%)	H_2_O influx (ml/kg/day)	CO_2_ (ml/g/hr)		FMR (W)		FMR (W/kg)	
									Nagy	Speakman	Nagy	Speakman	Nagy	Speakman
San Nicolas														
C2	85.4	81.2	Y	22.2	7.2	4.1	65.0	174	0.449	0.233	245.3	127.0	2.94	1.52
C3	78.0	75.4	Y	‐	8.1	4.2	64.7	154	0.763	0.545	383.7	274.2	5.00	3.58
C8	59.8	63.4	Y	13.0	9.0	6.6	66.6	172	0.997	0.743	402.5	300.4	6.53	4.88
C12	78.4	79.4	U	‐	17.1	11.0	47.5	129	1.045	0.836	540.6	432.6	6.85	5.48
C14	86.2	76.0	U	‐	11.9	8.4	64.0	83	0.774	0.628	411.5	334.1	5.07	4.12
C16	94.2	87.0	Y	‐	14.1	8.8	63.5	130	0.880	0.678	522.7	402.9	5.77	4.45
C18	86.0	84.8	Y	‐	11.1	7.3	65.1	139	0.713	0.510	399.1	285.6	4.67	3.34
C20	82.8	76.2	U	‐	10.1	4.8	62.1	77	0.544	0.420	283.4	219.0	3.56	2.75
C22	95.4	83.2	Y	8.3	11.2	7.3	63.0	127	0.969	0.763	567.4	446.7	6.35	5.00
San Miguel														
WAF2001	84.5	72.0	Y	19.0	3.8	2.2	63.2	85	0.664	0.527	340.8	270.2	4.35	3.45
WAF2002	85.2	79.8	Y	13.0	9.9	7.1	62.9	142	0.606	0.415	327.7	224.3	3.97	2.72
WAF2007	81.8	80.2	Y	14.8	5.9	4.9	63.6	150	0.791	0.578	420.2	306.7	5.19	3.79
WAF2010	75.6	74.0	Y	16.6	4.8	2.8	64.0	124	0.756	0.571	370.6	279.9	4.95	3.74
WAF2018	86.9	79.6	Y	19.8	10.8	5.4	64.4	122	0.662	0.486	361.5	265.3	4.34	3.18
WAF2025	78.9	77.9	Y	17.2	8.1	5.8	63.4	170	0.855	0.614	439.5	315.7	5.61	4.03

Pup refers to whether a female was observed nursing a pup (Y) or whether her pup status was unknown (U).

CO_2_ production and FMR were calculated using equations from Nagy (1980) and Speakman et al. (1993).

**Table 2:** At‐sea field metabolic rates (FMR) for adult female California sea lions and the behavioral variables used in the principal components regression. Values for behavioral variables represent mean values for all dives or bouts across the foraging trip

Sea lion ID	At‐sea FMR (W/kg)		Depth (m)	Duration (s)	Ascent rate (m/s)	Descent rate (m/s)	Bout duration (hr)
	Nagy	Speakman					
Mixed							
C2	5.30	3.29	54.5	119.3	1.18	1.11	1.3
C8	8.00	5.98	93.3	185.0	1.50	1.63	1.5
C12	8.84	6.97	130.5	226.2	1.44	1.53	1.9
C14	6.72	5.35	39.0	142.1	1.15	1.32	2.0
C16	7.85	6.01	143.2	225.8	1.49	1.53	1.2
C20	6.49	4.95	42.1	132.3	1.24	1.43	1.4
C22	8.27	6.44	92.2	173.3	1.45	1.44	1.7
WAF2001	6.70	5.21	32.0	122.1	1.13	1.21	1.2
WAF2002	5.58	3.93	74.1	202.3	1.17	1.33	1.6
WAF2010	7.26	5.47	82.4	167.7	1.23	1.31	1.7
WAF2025	7.18	5.20	41.9	157.2	1.07	1.06	1.3
Deep							
C3	7.29	5.29	161.7	238.0	1.44	1.61	1.8
C18	6.56	4.75	188.9	255.5	1.45	1.59	2.5
WAF2007	6.15	4.51	203.0	265.2	1.54	1.63	3.3
WAF2018	7.10	5.25	105.2	169.6	1.02	1.00	1.1

At‐sea FMR was calculated using equations from Nagy (1980) and Speakman et al. (1993).

**Figure 1:**
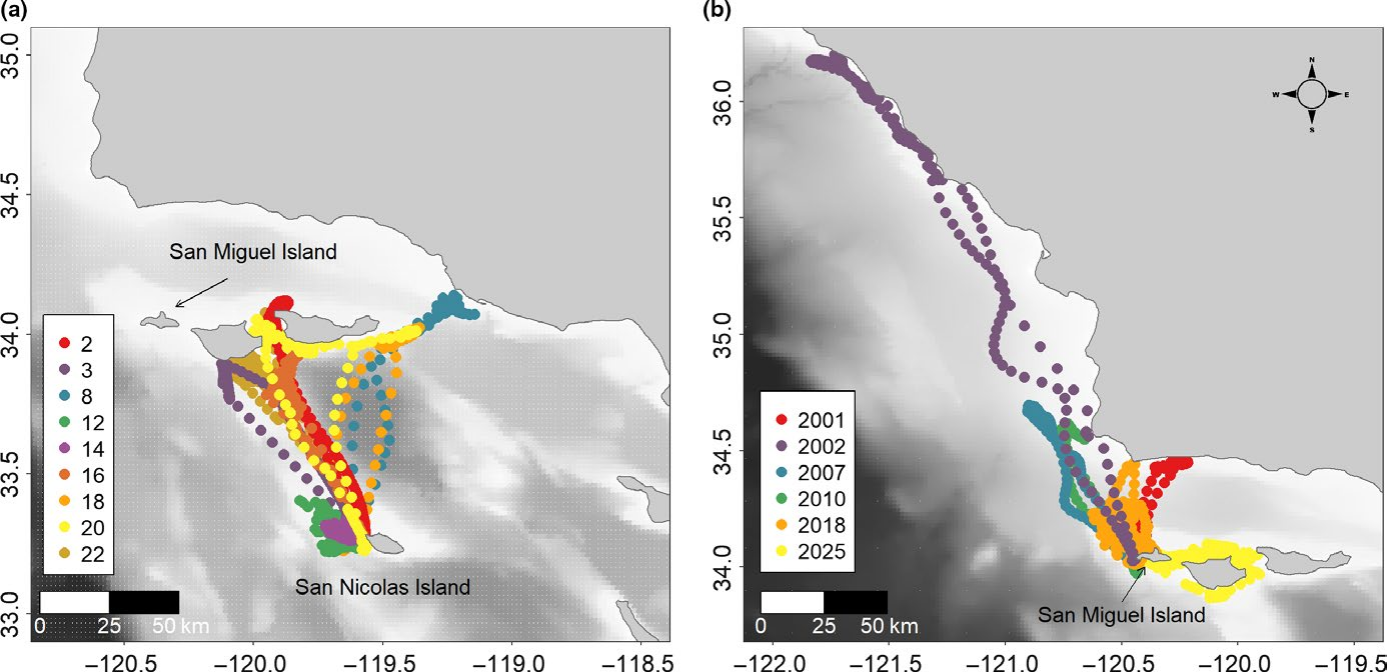
Interpolated at‐sea locations of adult female California sea lions from San Nicolas Island (a) and San Miguel Island (b) plotted over bathymetry

Sea lions from SNI primarily foraged around the northern Channel Islands, whereas the majority of sea lions from SMI foraged north of the Channel Islands along or just off the mainland coast (Figure 1). The majority of foraging trips fell into one of two foraging strategies, a mixed strategy consisting primarily of benthic and epipelagic dives (31 of 44 trips), and a deep‐diving strategy consisting primarily of deep epipelagic and mesopelagic dives (11 of 44 trips; Figure S3). Sea lions that undertook multiple foraging trips to sea generally had similar behavior on all trips and clearly had one dominant foraging strategy. Overall, 11 sea lions were classified as mixed strategy foragers, whereas the remaining four sea lions were classified as deep‐diving foragers.

**Figure 2:**
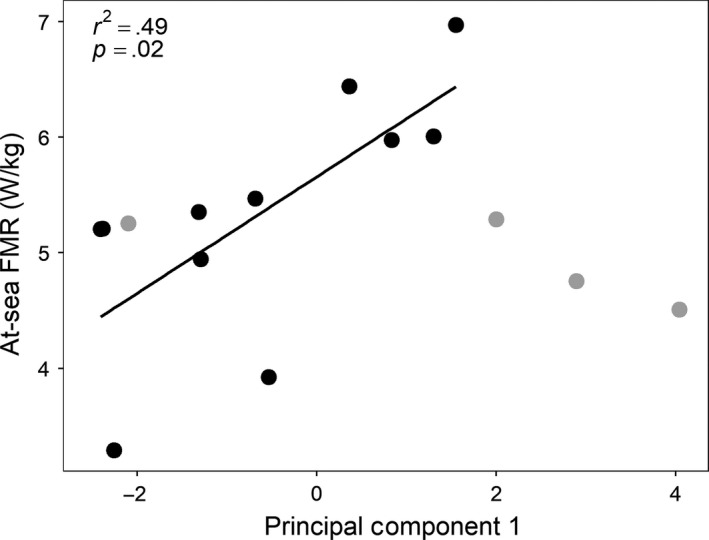
The relationship between at‐sea field metabolic rate (FMR) and the first principal component for adult female California sea lions that used a mixed foraging strategy. Sea lions that used the deep‐diving strategy are shown in gray for comparison. The three behavioral variables that loaded strongly (>0.3) on the first component were dive depth (0.54), dive duration (0.52), and bout duration (0.66)

**Figure 3:**
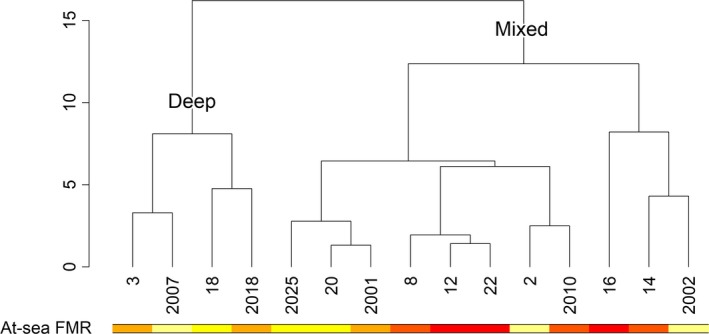
Dendrogram of distance matrix of the first three principal components from the foraging strategy analysis showing how individual sea lions clustered together in multivariate space with respect to fine‐scale variation in foraging behavior. The color bar represents at‐sea FMR, with values ranging from low (yellow) to high (red). There was no correlation between behavioral and energetic distance matrices, illustrated here by the lack of distinct color clusters

The first two principal components explained 90% of the variance in the data and were used in regressions with at‐sea FMR. There was a significant interaction between the first principal component axis and foraging strategy (*p *=* *.05), thus the relationship between at‐sea FMR and the first principal component was evaluated separately for each foraging strategy. The first principal component explained a significant amount of variation in at‐sea FMR for mixed strategy (*r* ^2^ **=* *.49, *p *=* *.02) but not deep‐diving foragers (*r* ^2^ **=* *.57, *p *=* *.24; Figure 2). The variables that loaded strongly on this axis were mean dive depth (0.54), dive duration (0.52), and bout duration (0.66). There was no relationship between at‐sea FMR and the second principal component (*r* ^2^ **=* *.20, *p *=* *.09) where the remaining two variables, ascent and descent rate, loaded strongly. With regards to fine‐scale variation, three of the four sea lions with the highest at‐sea FMRs clustered together behaviorally; all three of these sea lions had much greater day diving depths compared with most of the other sea lions using the mixed foraging strategy. There was however no correlation between distance matrices of at‐sea FMR and PCA scores from the foraging strategy analysis (*r *=* *−.16, *p*_sim_ = 0.81). This did not change if separate correlations were performed for each foraging strategy, indicating that sea lions with similar fine‐scale behavior did not necessary have similar energy expenditure (Figure 3).

**Figure 4:**
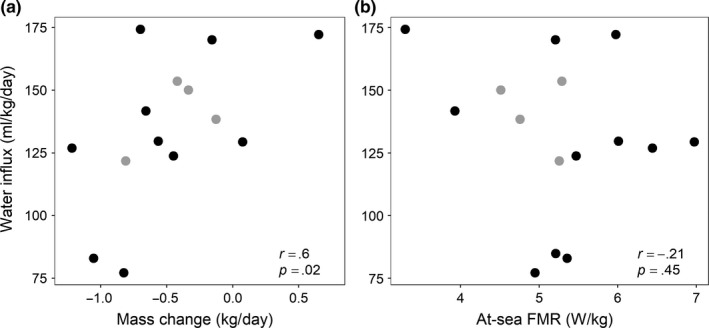
Correlations between water influx rates and mass change (a) and at‐sea field metabolic rate (FMR; b) for 15 adult female California sea lions. Colors represent sea lions that used different strategies on their foraging trip to sea (mixed foraging strategy (black) or deep‐diving strategy (gray))

Water influx rates ranged from 77.2 to 174.3 ml kg^−1^ day^−1^ with mean values that were similar between islands (SNI* *=* *131.7 ± 34.2 ml kg^−1^ day^−1^, SMI = 132.1 ± 29.2 ml kg^−1^ day^−1^; two‐way ANOVA, *F*_1,11_ < 0.01, *p *=* *.96) and foraging strategies (mixed* *=* *141.0 ± 14.3 vs. deep* *=* *128.5 ± 35.4 ml kg^−1^ day^−1^; two‐way ANOVA, *F*_1,11_ = 0.10, *p *=* *.55). Water influx rate and the rate of mass change were positively correlated (*r *=* *.60, *p *=* *.02, Figure 4a), but there was no correlation between at‐sea FMR and water influx rates (*r *=* *−.21, *p *=* *.46, Figure 4b).

## 4 DISCUSSION

### 4.1 Influence of behavior on at‐sea FMR

Adult female California sea lions exhibited considerable variability in at‐sea FMR, with some individuals spending energy at almost twice the rate of other individuals. For an 80 kg sea lion, this difference amounts to consuming an additional 3.5–6 kg of prey per day depending on the energy density of common prey species. With the exception of Galapagos sea lions (*Z. wollebaeki*), this variability in energy expenditure while at sea appears to be relatively common in otariids, as northern fur seals (*Callorhinus ursinus*), Antarctic fur seals (*Arctocephalus gazella*), Australian sea lions (*Neophoca cinerea*), and New Zealand sea lions (*Phocarctos hookeri*) all exhibited a similar magnitude of ranges in at‐sea FMR (Costa, Croxall, & Duck, 1989; Costa & Gales, 2000, 2003; Costa & Gentry, 1986; Villegas‐Amtmann, McDonald, Páez‐Rosas, Aurioles‐Gamboa, & Costa, 2017). Despite this similarity, California sea lions exhibited considerably more variability in at‐sea behavior than these temperate and Arctic species, exploiting prey at a wide range of depths within the water column and at or near the seafloor. This variation in at‐sea behavior of California sea lions did explain some of the variability in at‐sea FMR, although the influence of behavior was affected by whether sea lions used the mixed or deep‐diving foraging strategy.

The first principal component explained 49% of the variation in at‐sea FMR for sea lions that used the mixed foraging strategy, which was predominantly due to variation in dive depth, dive duration, and bout duration among sea lions. Although all three variables may have influenced at‐sea FMR, dive depth may have been the primary driver given that (1) dive depth has an influence on dive duration but the opposite is not necessarily true (i.e., it takes more time for sea lions to reach deeper depths but they can have long duration dives that are irrespective of depth), and (2) our initial exploratory plots between at‐sea FMR and each individual variable showed no apparent relationship between the bout duration of mixed strategy foragers and at‐sea FMR (i.e., bout duration appeared more influential for deep‐diving sea lions). The finding that at‐sea FMR increased with dive depth was unexpected given a previous study that found the opposite relationship (Costa & Gales, 2000) and because of energy‐saving swim strategies associated with changes in buoyancy that air‐breathing marine predators use on deeper dives, such as stroke‐and‐glide swimming (Crocker, Gales, & Costa, 2001; Tift, Hückstädt, McDonald, Thorson, & Ponganis, 2017; Watanuki, Niizuma, Gabrielsen, Sato, & Naito, 2003; Williams et al., 2000). Tift et al. (2017) found that adult female California sea lions primarily used passive swimming strategies on dives >100 m, which comprised between <0.1% and 63% of all dives for each sea lion using the mixed foraging strategy. While mixed foragers may experience some cost savings associated with passive swimming strategies on deeper foraging dives, it appears that they are overshadowed by other behaviors on deeper dives that result in increased energy expenditure.

For California sea lions, we suspect that changes in dive depth are related to changes in prey type or prey age class, such that the sea lions using the mixed strategy with higher at‐sea FMRs targeted (or spent more time targeting) prey that were costly to find or capture. McHuron et al. (2016) hypothesized that sea lions using this foraging strategy may target species that are both demersal and pelagic, such as market squid (*Doryteuthis opalescens*) and Pacific hake (*Merluccius productus*), and scats collected from these and other instrumented sea lions at San Miguel during our study support this hypothesis (Marine Mammal Laboratory, unpublished data). Market squid are abundant in the northern Channel Islands from October to March and are primarily found within the 100 m isobath where the majority of commercial fishing occurs (Maxwell et al., 2004; Zeidberg et al., 2012). Juvenile hake are typically distributed within the water column and are frequently consumed by adult female California sea lions, whereas adult hake are found at or near the bottom at depths corresponding to the continental slope (Bailey, Francis, & Stevens, 1982) and are not targeted as frequently as juvenile age classes (Orr, VanBlaricom, DeLong, Cruz‐Escalona, & Newsome, 2011). Scat samples collected from instrumented females indicated that adult females consumed adult hake during our study period and in the several months following, and the at‐sea locations of the four sea lions in the mixed strategy group with the highest at‐sea FMRs were generally consistent with foraging over the continental slope. Thus, the positive relationship between at‐sea FMR and depth may result from a stronger dependence on adult hake that are more energetically expensive to capture than juvenile hake and market squid found at shallower depths and/or within the water column.

Behavioral variation appeared to have different energetic consequences for California sea lions depending on foraging strategy, as indicated by the significant interaction between the first principal component and foraging strategy. This may be due to some differences in prey types, such as mesopelagic fishes (McHuron et al., 2016), but also may reflect a greater dependence on passive swimming strategies and differences in oxygen management between the two foraging strategies, including a pronounced dive response and lung collapse at deeper depths (Kooyman & Ponganis, 1998; McDonald & Ponganis, 2012). McDonald and Ponganis (2014) found the dive response, characterized by extreme bradycardia (<10 beats/min), of California sea lions was pronounced on longer dives (68% of dives >4 min and 98% of dives >5 min), but more variable on short dives (<3 min) where only 43% of dives had heart rates below resting. Similarly, blood flow to swimming muscles appears to be restricted during dives >100 m but is not consistently regulated during shallower dives in this species (Tift et al., 2017). Sea lions using the deep‐diving strategy generally had a much greater percentage of long duration dives, with an average of 51% of dives >4 min and 46% >5 min compared with mixed strategy foragers that only had an average of 24% of dives >4 min and 14% >5 min. They also had a much greater percentage of dives to depths >200 m than mixed strategy foragers (46% vs. 12.5%), which is the depth associated with complete lung collapse in this species (McDonald & Ponganis, 2012). Collectively, this suggests that behavioral and physiological mechanisms that conserve oxygen on long, deep dives may affect the relationships between energy expenditure and variation in diving behavior. We did not detect any relationships between at‐sea FMR and the two principal components for sea lions using the deep‐diving strategy, which may have been due to a small sample size, the mitigating effects of oxygen management strategies on variation in at‐sea FMR, or because the behaviors we measured truly did not influence energy expenditure.

It is important to recognize that there was considerable variation in at‐sea FMR that was unexplained by individual variation in behavior, particularly for the 11 sea lions using the mixed foraging strategy. The remaining unexplained variability could have been due to a variety of intrinsic factors, such as individual differences in resting metabolic rates (Broggi et al., 2007; Larivée, Boutin, Speakman, McAdam, & Humphries, 2010), differences in foraging efficiency due to experience (Hoskins, Costa, Wheatley, Gibbens, & Arnould, 2015; McDonald, Goebel, Crocker, Tremblay, & Costa, 2009), or individual variation in maternal investment (McDonald, Goebel, Crocker, & Costa, 2012). It also is possible that we were unable to capture important predictor variables using the methods employed in this study, as we only measured two‐dimensional behavior. The use of additional bio‐logging devices, such as 3‐axis accelerometers and video cameras, can be attached to many large predators and would be helpful in further elucidating behaviors that may affect energy expenditure, particularly as it relates to overall body movement and diet.

### 4.2 Influence of foraging strategy on at‐sea FMR and prey intake

There were no differences in the mean at‐sea FMR or water influx rates between sea lions using the mixed and deep‐diving strategies, suggesting there were no clear energetic benefits associated with using one particular foraging strategy. It is likely however that the energetic cost of a foraging strategy is dynamic through time given that at‐sea FMR is affected by diving behavior, which varies due to temporal variation in the distribution of prey species (Kuhn & Costa, 2014; Melin, DeLong, & Siniff, 2008). Our findings regarding the energetic trade‐offs between foraging strategies should therefore not be extrapolated beyond our study year without further measurements. Our data were not consistent with the hypothesis that benthic diving is energetically expensive (Costa et al., 2004), as indicated by the similarity in mean at‐sea FMRs between the two foraging strategies and the lack of a correlation between behavioral and energetic similarity. It may be that diving to or near the sea floor is not itself inherently energetically costly, but that the expense is driven by the specific behaviors undertaken while at depth. Thus, there may not be clear intra‐ or interspecific trends for otariids because different individuals or species may expend variable amounts of energy on benthic dives depending on the behaviors used and prey types that are pursued.

While there was a lack of energetic trade‐offs between foraging strategies, sea lions using the deep‐diving strategy neither lost nor won big in terms of energy balance, as evidenced by the smaller and intermediate ranges of at‐sea FMRs, water influx rates, and mass changes compared with mixed strategy foragers. This pattern deserves further exploration because it may be an indication that foraging strategies differ in the variability of energy balance that an individual sea lion experiences.

### 4.3 Balancing energy expenditure and gain

There was no evidence that sea lions that expended energy at a higher rate had a greater rate of prey intake, which is contradictory to what we would expect based on predictions of optimal foraging theory. Water influx rate is not synonymous with prey or energy intake when the diet is unknown (Costa, 1987), but the positive correlation with the daily rate of mass change for sea lions suggests that higher rates of water influx were reflective of greater prey intake and thus likely greater energy intake. Previous studies have found variable results, with positive relationships detected for some but not all otariid species (Arnould et al., 1996; Costa, 2008; Costa & Gales, 2000, 2003; Costa & Gentry, 1986; Costa et al., 1989; Villegas‐Amtmann et al., 2017). Pup masses and growth rates from SMI were below average in 2014 (Leising et al., 2015), and evidence from our study supports the hypothesis that foraging conditions in southern and central California were unfavorable for sea lions. The majority of our sea lions lost mass across the measurement period, which is atypical of these types of studies (Arnould et al., 1996; Costa & Gales, 2000, 2003; Costa & Gentry, 1986; Costa et al., 1989, 1991). Although most sea lions were still nursing a pup, their 6‐month‐old pups weighed considerably less than the long‐term average of 7‐month‐old pups (16 vs. 25–28 kg, S. Melin unpublished) and there was large variation in pup mass among females. Consequently, the lack of a positive relationship between at‐sea FMR and water influx may reflect the difficult foraging conditions encountered by sea lions that were balancing reproductive and maintenance costs during unfavorable conditions. The wide range of pup masses indicated there was individual variation in the amount of energy being delivered to pups through lactation. Differential maternal investment could explain some of the variability in energy expenditure/prey intake among sea lions (i.e., small pups require less energy than large pups), but also may reflect that higher effort did not yield greater prey yield and that this energy deficit was transferred to the pup (i.e., sea lions may work harder when they are unsuccessful).

## 5 CONCLUSIONS

Intraspecific variation in behavior has clear energetic consequences for female California sea lions, which has implications for reproductive success and population dynamics. Variables that integrated behavior across the entire foraging trip were important predictors of energy expenditure but foraging strategy was not; however, the classification of individuals to a foraging strategy was important in elucidating the behaviors that influenced energy expenditure. Although sea lions have the flexibility to switch foraging strategies, they generally use one dominant strategy and exhibit consistency in their mean dive depth and duration across foraging trips, at least at short temporal scales (1–2 months; McHuron, 2016). During our study period, pups were still dependent on their mothers for nutrition and therefore, their growth was dependent on energy obtained on each foraging trip by their mothers. Consequently, even short‐term increases in energy expenditure could negatively impact pup growth, particularly as these increases are not necessarily offset by increases in energy gain for the mother.

There appears to be an upper limit on the range in at‐sea FMRs exhibited by otariids, which may enable California sea lions to function in a dynamic environment by constraining energy expenditure while allowing for the exploitation of prey at a wide range of depths and habitats. We found relationships between energy expenditure and behavior that differed not only between foraging strategies but also with studies on other otariid species, highlighting the need to be cautious in extrapolating findings without a better understanding of the mechanisms driving these relationships. We hypothesize that the influence of behavioral variation on energy expenditure of California sea lions is modulated by interactions between prey type and physiological mechanisms for oxygen conservation, but further study is needed to provide support for this hypothesis.

The results from our study underscore the need to quantify the energetic consequence of behavioral variation for large carnivores, especially given the disproportionate influence of carnivores on ecosystem dynamics. These types of studies have primarily been limited to a small range of species, but technological advances and pairing of data from captive individuals and wild populations make it increasingly possible to quantify these relationships for large carnivores.

## CONFLICT OF INTEREST

None declared.

## DATA ACCESSIBILITY

All data are presented in text or as Supplemental Material.

## AUTHOR CONTRIBUTIONS

DPC and EAM conceived the ideas and designed the methodology. EAM, SHP, LAH, SRM, and JDH collected the data. EAM analyzed the data and led the writing of the manuscript. All authors contributed critically to the drafts and gave final approval for publication.
