# Supplementary material for: The energetic consequences of behavioral variation in a marine carnivore
Source: Ecol Evol. 2018 Apr 2;8(8):4340–51. doi: 10.1002/ece3.3983 (PMC5916299; doi:10.1002/ece3.3983)
Supplement: Supplementary file 1 [file ECE3-8-4340-s001.docx]

**Supplementary Materials**

**The energetic consequences of behavioural variation in a marine carnivore**

Elizabeth A. McHuron^a*^, Sarah H. Peterson^a,b^, Luis A. Hückstädt^a^, Sharon R. Melin^c^, Jeffrey D. Harris^c^, and Daniel P. Costa^a^

^a^Department of Ecology & Evolutionary Biology, University of California, Santa Cruz, CA, USA

^b^Institute of Marine Sciences, Long Marine Laboratory, Santa Cruz, CA, USA

^c^Marine Mammal Laboratory, Alaska Fisheries Science Center/NOAA, Seattle, WA, USA

*Corresponding author: emchuron@ucsc.edu

Pages: 9

Tables: 2

Figures: 3

**Description of methods and results of calculation of variability in CO_2_ production and at-sea FMR estimates**

We estimated the variability in our estimates of at-sea FMR by recalculating CO_2_ production for each female by mixing and matching individual triplicate measurements from the background, equilibration, and final serum samples (Speakman 1997). The mean and SD of these estimates were calculated from 10,000 simulations and used as an indication of the precision of our estimate of CO_2_ production. For each simulation, we also calculated at-sea FMR for each sea lion as described in the main text as an indication of the precision of our estimate of at-sea FMR used in statistical analyses.

The variability in CO_2_ production values for each animal from simulations was very small (SD ≤ 0.01), and mean CO_2_ production values from simulations were within 0.0005 ml g^-1^ hr^-1^ of values used to calculate FMR. The variability among females in background isotope levels was also relatively small; background D^2^ values differed by 0.32 ppm and O^18^ by 0.48 ppm. This resulted in simulation at-sea FMR estimates that were very similar to actual values used in the analysis (Figure S1). Minimum values of at-sea FMR from the simulations were on average 0.21 W kg^-1^ (0.14 - 0.28) lower than actual values, and maximum values were on average 0.07 W kg^-1^ higher than actual values.

One female from SNI (C12) had isotope concentrations at recapture that were above, but close to background levels. This can result in erroneous estimates of energy expenditure if there is significant error in estimates of CO_2_ production, or considerable variability in background isotope levels between the initial capture and recapture (background levels at recapture are assumed to be the same as initial). As presented above, the variability in CO_2_ production values and estimates of at-sea FMR from these values was relatively small for each animal. In addition, the variability among females in background isotope levels was also relatively small, and C12 had enrichment levels higher than this variability in background isotope levels at the time of recapture. We therefore chose to include C12 in statistical analyses because (1) we were confident in our estimate of CO_2_ production and at-sea FMR, (2) the slight variations in background isotope values between the initial and recapture would not have significantly affected the magnitude of the estimate (i.e., a high value would still have been a high value), and (3) inclusion of this sea lion in statistical analyses did not affect any of the overall trends.


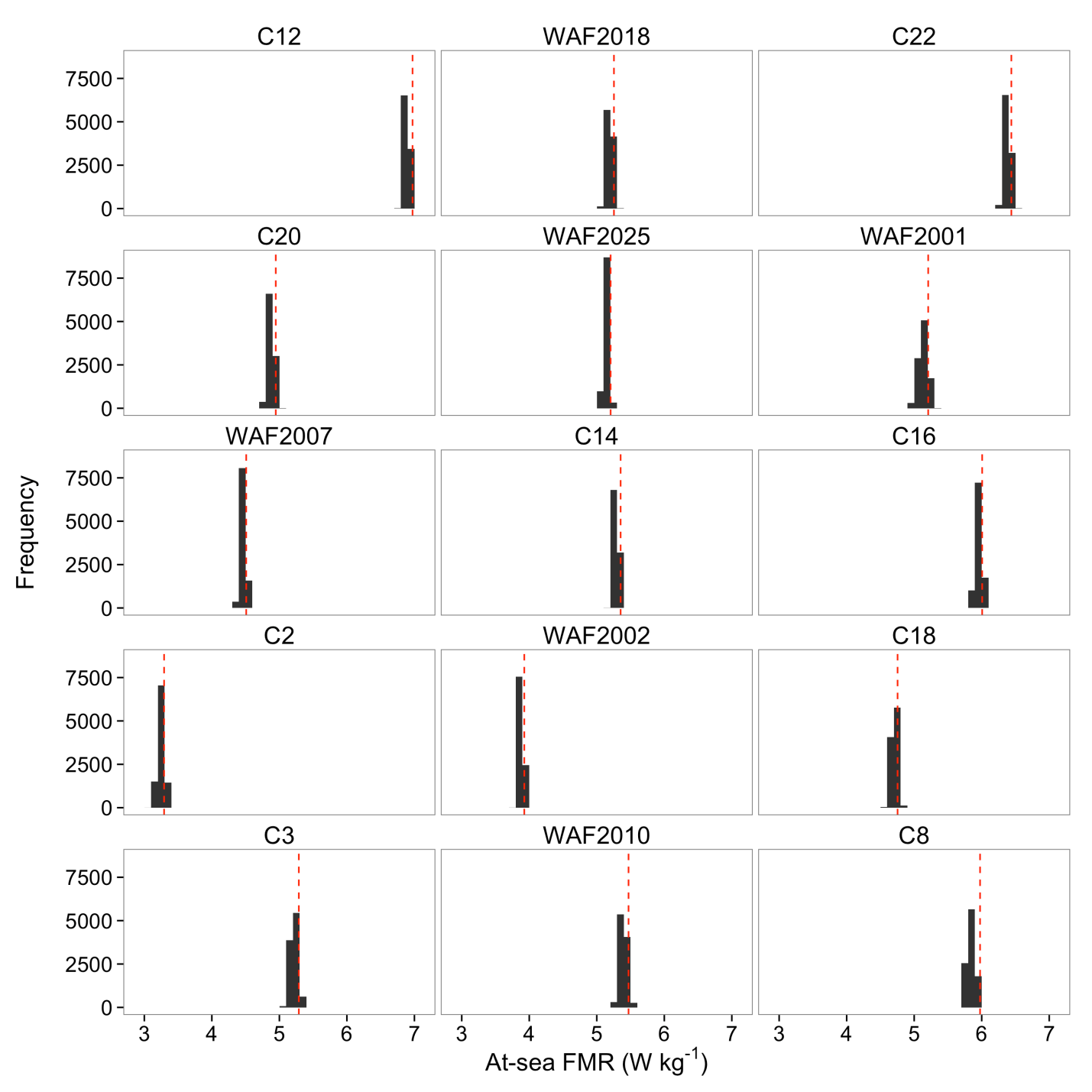


Figure S1. Distribution in at sea FMR estimates (W kg^-1^) obtained by simulations that combined individual triplicate measurements from the background, equilibration, and final serum samples. Each subplot represents an individual sea lion, with their identification number denoted at the top of the subplot. The red dashed line in each subplot represents the actual value of at-sea FMR used in statistical analyses, which was calculated from mean values of triplicate measurements at each time period.

**
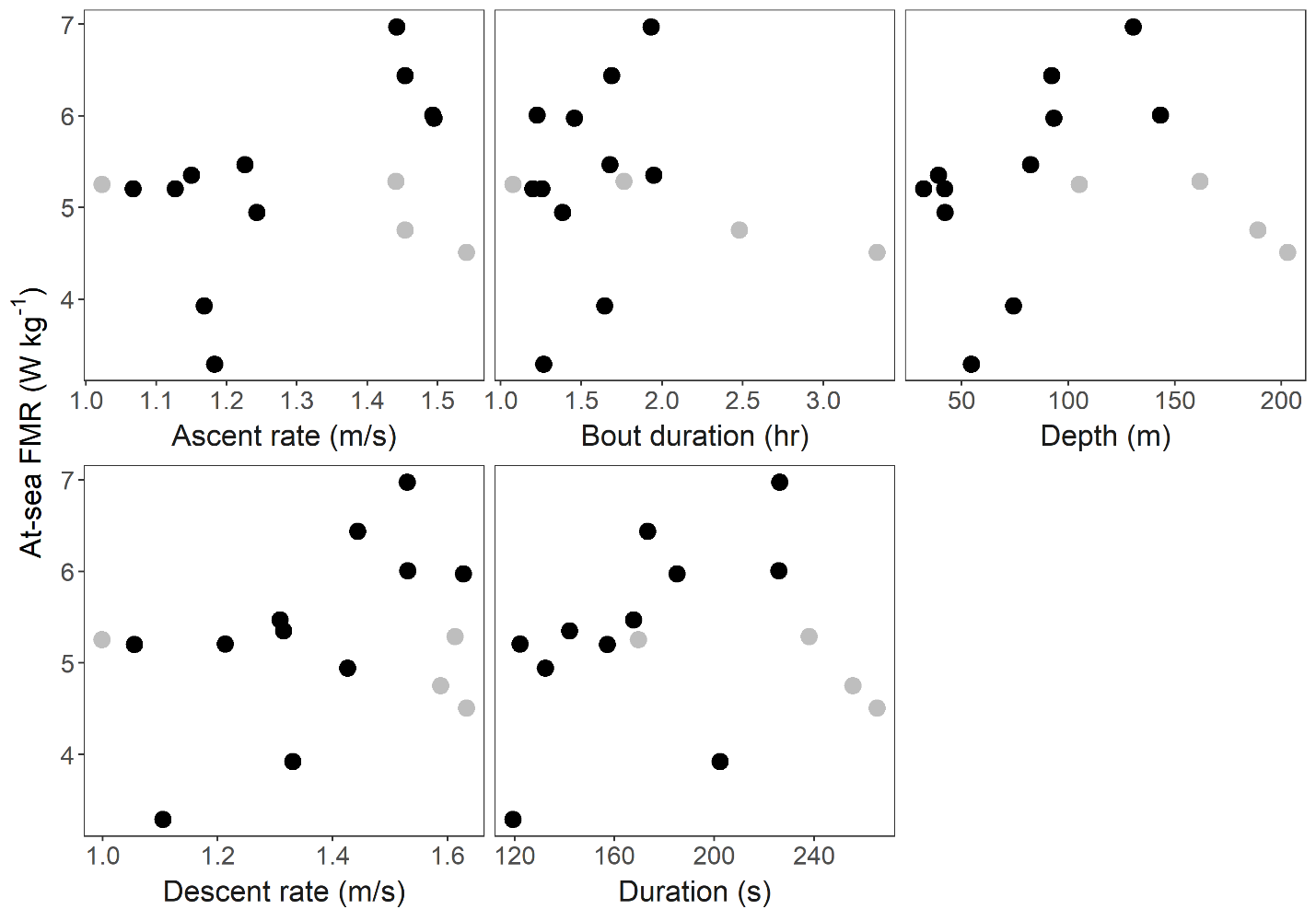
**

Figure S2. The relationship between at-sea field metabolic rate (FMR) and each of the five variables used in the principal components analysis. Each point is colored based on the foraging strategy that female used on her foraging trip/s (black = mixed strategy, grey = deep).


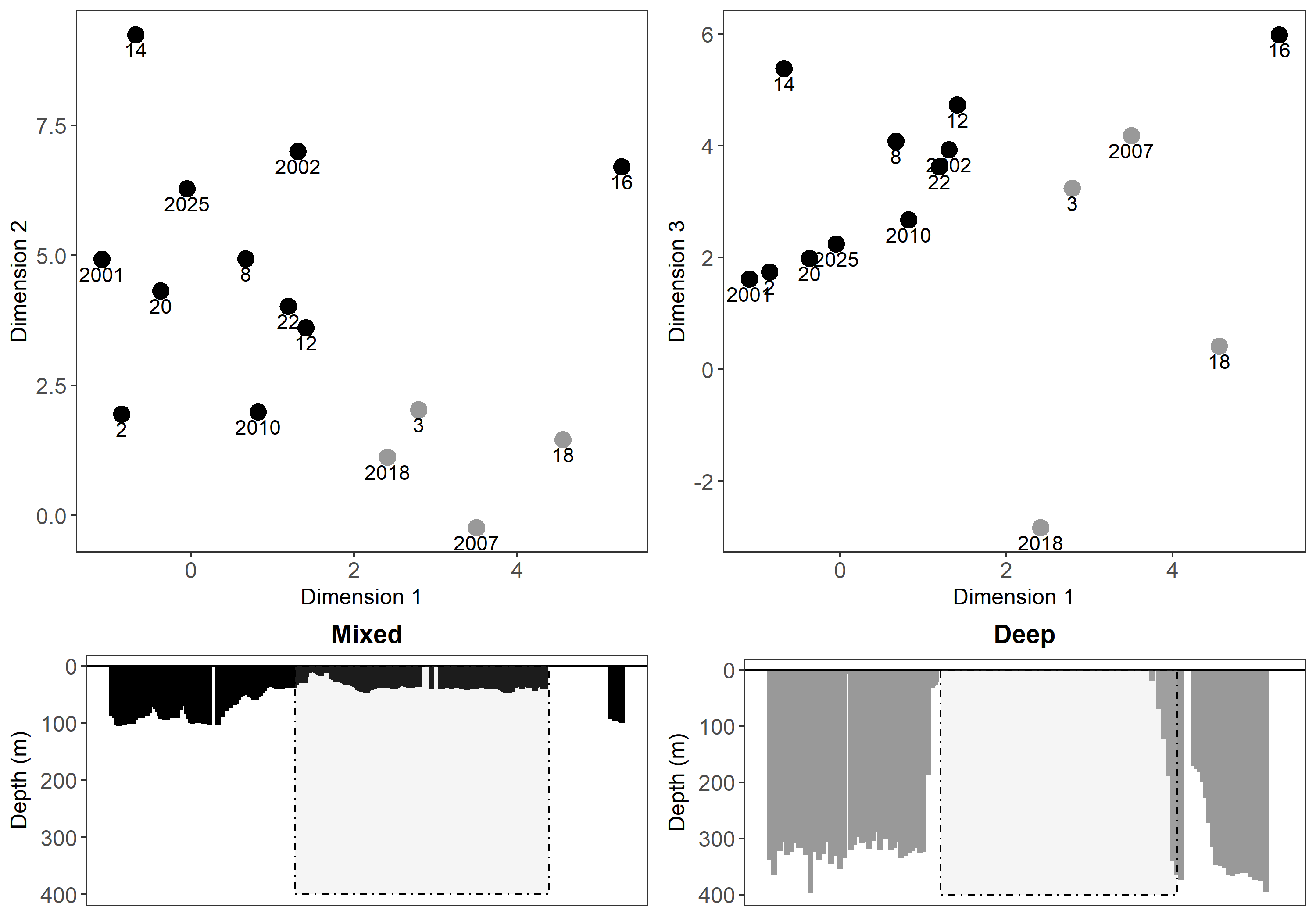


Figure S3. Relationships between scores from the principal components analysis (PCA) describing the variation among sea lions in diving behaviour (top panel). PCA scores from the first three dimensions were used to identify the foraging strategy of each sea lion. Dimension 1 primarily describes variation related to benthic diving, while Dimensions 2 and 3 primarily describe differences related to dive depth and day/night behaviour. Each point represents a single foraging trip or the mean values of all foraging trips for sea lions that had more than one trip to sea. Points are colour-coded by the foraging strategy to which each sea lion was classified and labelled with the animal identification number. The bottom panel shows the dive profile from a 24-hour period of two sea lions, one whose foraging trip was classified to the mixed foraging strategy (Mixed) and the other that was classified to the deep-diving foraging strategy (Deep). Local night is shown by the shaded box in each plot.

Table S1. Diving variables considered as explanatory variables of at-sea FMR of adult female California sea lions.

| Variable | Description or Equation |
| --- | --- |
| Ascent rate (m s^-1^)^a^ | The time to reach the bottom of the dive from the surface |
| Descent rate (m s^-1^)^a^ | The time to reach the surface from the bottom portion of the dive |
| Depth (m)^a^ | Maximum depth of each dive |
| Duration (s)^a^ | The total duration of each dive |
| Bottom time (s)^a^ | The amount of time spent at 80% or greater of the maximum dive depth |
|  |  |
| Dive effort (m min hr^-1^) | $\frac{\sum_{i}^{n} 2*depth_{i}*duration_{i}}{TimeatSea}$  Where n is the total number of dives and *TimeatSea* is the total time at sea (Bowen *et al.* 2001) |
| Post-dive interval (s)^a^ | The time between the end of one dive and the start of the next dive |
| Vertical transit rate (km day^-1^) | The sum of the vertical distance travelled underwater divided by the time spent at sea |
| Bout duration (hr)^b^ | The time from the start of the first dive in a bout to the end of the last dive in the bout |
| Dive rate (# dives hr^-1^) | The number of dives per hour |
| Bout interval:bout duration^b^ | Ratio between the end of one bout and the start of the next bout and the total duration of each bout |
| Inter-bout interval (hr)^b^ | The time between the end of one bout and the start of the next bout. Bouts that ended and were followed by a period of time spent hauled-out were excluded from this calculation |
| Time at bottom of dive (%) |  |
| Time diving (%) |  |

^a^Values were calculated for each individual dive and then averaged across all dives to obtain a mean value.

^b^Dive bouts are periods of intensive diving activity and were identified as described in McHuron *et al.* (2016).

Table S2. Diving variables used in the classification of foraging trips to one of the three foraging strategies used by adult female California sea lions.

| Variable | Description or equation |
| --- | --- |
| Day depth (m)^a^ | Maximum depth of dives that occurred during the day (solar zenith < 90) |
| Night depth (m) ^a^ | Maximum depth of dives that occurred at night (solar zenith > 102) |
| Bottom time (s) ^a^ | The amount of time spent at 80% or greater of the maximum dive depth |
| Bottom wiggles ^a^ | The number of vertical excursions at the bottom of the dive |
| Intra-depth zone index (IDZ) ^a^ | A measure of the tendency to dive repeatedly to a given depth (Tremblay & Cherel 2000) |
| Diel | A measure of the difference between day and night diving depths, calculated as $\frac{Depth_{day}-Depth_{night}}{\max(Depth_{day},Depth_{night})}$ |
| Efficiency ^a^ | $\frac{BottomTime}{Duration+Post\text{ }Dive\text{ }Interval}$ |
| Time diving (%) |  |
| Day diving (%) |  |
| Night diving (%) |  |
| Epipelagic (%)^b^ |  where epipelagic dives (Epi) were classified as dives< 200 m that were not at or near the seafloor |
| Benthic (%)^b^ |  where benthic dives were dives classified as being at or near the seafloor |
| Mesopelagic (%)^b^ |  where mesopelagic dives (Meso) were classified as dives ≥200 m that were not at or near the seafloor |

^a^Values were calculated for each individual dive and then averaged across all dives to obtain a mean value

^b^A complete description of how dives were classified into the three categories (epipelagic, benthic, and mesopelagic) is presented in McHuron *et al.* (2016)

**Literature Cited**

Bowen, W.D., Iverson, S.J., Boness, D.J. & Oftedal, O.T. (2001) Foraging effort, food intake and lactation performance depend on maternal mass in a small phocid seal. *Functional Ecology*, **15**, 325–334.

McHuron, E.A., Robinson, P.W., Simmons, S.E., Kuhn, C.E., Fowler, M. & Costa, D.P. (2016) Foraging strategies of a generalist marine predator inhabiting a dynamic environment. *Oecologia*, **182**, 995–1005.

Speakman, J.R. (1997) *Doubly Labelled Water: Theory and Practice*. Chapman & Hall, London, UK.

Tremblay, Y. & Cherel, Y. (2000) Benthic and pelagic dives: a new foraging behaviour in rockhopper penguins. *Marine Ecology Progress Series*, **204**, 257–267.
